# Do the pre-service education programmes for midwives in India prepare confident ‘registered midwives’? A survey from India

**DOI:** 10.3402/gha.v8.29553

**Published:** 2015-12-07

**Authors:** Bharati Sharma, Ingegerd Hildingsson, Eva Johansson, Malvarappu Prakasamma, K.V. Ramani, Kyllike Christensson

**Affiliations:** 1 Department of Women's and Children's Health, Karolinska Institutet, Solna, Sweden; 2 Centre for Management of Health Services, Indian Institute of Management, Ahmedabad, India; 3 Department of Nursing, Mid Sweden University, Sundsvall, Sweden; 4 Department of Women's and Children's Health, Uppsala University, Uppsala, Sweden; 5 Global Health, Karolinska Institutet, Solna, Sweden; 6 Academy of Nursing Studies and Women's Empowerment Research, Hyderabad, India

**Keywords:** midwifery skills, confidence, nurses, India

## Abstract

**Objective:**

The graduates of the diploma and degree programmes of nursing and midwifery in India are considered skilled birth attendants (SBAs). This paper aimed to assess the confidence of final-year students from pre-service education programmes (diploma and bachelor's) in selected midwifery skills from the list of midwifery competencies of the International Confederation of Midwives (ICM).

**Design:**

A cross-sectional survey was conducted in Gujarat, India, involving 633 final-year students from 25 educational institutions (private or government), randomly selected, stratified by the type of programme (diploma and bachelor's). Students assessed their confidence on a four-point scale, in four midwifery competency domains – antepartum, intrapartum, postpartum, and newborn care. Explorative factor analysis was used to reduce skill statements into separate subscales for each domain.

**Results:**

Overall, 25–40% of students scored above the 75th percentile and 38–50% below the 50th percentile of confidence in all subscales for antepartum, intrapartum, postpartum, and newborn care. The majority had not attended the required number of births prescribed by the Indian Nursing Council.

**Conclusions:**

The pre-service education offered in the diploma and bachelor's programmes in Gujarat does not prepare confident SBAs, as measured on selected midwifery competencies of the ICM. One of the underlying reasons was less clinical experience during their education. The duration, content, and pedagogy of midwifery education within the integrated programmes need to be reviewed.

Skilled birth attendance (including midwifery) is crucial to saving lives and promoting health of women and newborn infants (1–4). Some studies seem to assume that the midwifery workforce across countries has uniform competence, scope of practice, and identity, which is regrettably not the case (5). Basic or core sets of competencies are needed to achieve the results described in these studies (6).

The International Confederation of Midwives (ICM) provides a list of the basic competencies expected from a pre-service midwifery education to qualify as a midwife, within the scope of practice defined by the ICM (7). The ICM recommends at least 18 months of midwifery education post-nursing or 3 years of direct entry education to achieve these sets of competencies. There are many pathways to midwifery found across countries: a direct entry curriculum, post-registration (nursing) curriculum, or embedded within a nursing curriculum (8).

India follows the integrated education pathway, having two formal pre-service education programmes for nursing and midwifery implemented across the country: a 3.5-year vocational training awarding a diploma by the schools of nursing and a university-based 4-year programme awarding a bachelor's degree by the nursing colleges. The time allotted to midwifery in both these programmes is about 11–19% (9, 10). All the graduates get a joint registration as registered nurse (RN) and registered midwife (RM). They get absorbed as general staff nurses in hospitals, expected to provide care under any medical speciality, one of them being maternity care.

To scale up skilled attendance at birth, India has introduced a 2- to 3-week in-service training for staff nurses to upgrade their midwifery skills (11), under the National Health Mission (12). In 2 to 3 weeks’ time, the skilled birth attendant (SBA) training is meant to develop competence of staff nurses in first-level care for obstetric and newborn complications (13). These staff nurses trained as SBAs are expected to provide independent maternity services in rural day and night primary health centres, and community health centres which are above the primary health centres.

There has been an ongoing discussion amongst health professionals, United Nations, and other bilateral organizations in India about the adequacy of the 2- to 3-week period for developing skills for managing complications. The government seems to assume that the staff nurses have the core sets of skills for attending normal labour. This assumption is questionable according to the some professionals in India (14).

## Confidence and competence

Concepts of confidence and competence may be linked but are not synonymous. *Confidence* as defined by the Oxford dictionary is ‘a feeling of self-assurance arising from an appreciation of one's own abilities or qualities’. *Competence* is, on the other hand, ‘the ability to do something successfully or efficiently’. Confidence has three influencing attributes: it is situational (depends on available time and resources), institutional (structure and pedagogy of educational programmes), and dispositional (personal traits, attitudes, and motivation) (15–18). Therefore, increased level of confidence may not be in direct proportion to increased competence, although decreased confidence may be linked to a reduction in skilled performance (19).

Poor performance and lack of confidence among newly qualified nurses was found to be a direct consequence of pre-registration programmes, lacking practice-based training (20, 21). According to Bandura (22), confidence is the strength of self-efficacy. Norman and colleagues found a number of factors which facilitated confidence, many of which emphasize student–teacher relationships, such as learning, experiencing, and achieving; feeling secure and receiving positive feedback; familiarity and receiving support and encouragement; working with staff at the teaching practice placement; and being treated well (15).

‘Self-efficacy’ or self-regulation is an important attribute of competency, having three dimensions: confidence, level (magnitude, or difficulty of tasks the individuals think they can perform), and generalizability (extent of transfer to other areas of functioning) (22). When applied to the midwife, confidence would concern task performance and perseverance when confronting difficulties and setbacks in the work situations. Butler et al. (23) identified ‘being a safe practitioner’ as one of the essential competencies required of a midwife at the point of registration. Being a safe practitioner included the ability to detect deviations and take appropriate action, and respond to emergency situations (24).

Given the facts that midwifery is part of nursing in India with much less duration than what is internationally recommended, we assumed that measuring confidence would partly reflect the quality of the teaching–learning experience the midwifery educational institutions are able to provide to their students and could also partially help to assess their fitness to practice as confident and competent midwives, post-registration.

This study aimed to assess the confidence of final-year students from the diploma and bachelor's programmes, in their skills in antepartum, intrapartum, postpartum, and newborn care from the list of midwifery competencies of the ICM.

## Method

This study reports findings of overall reported confidence of students against the four selected domains of ICM competencies. The comparison of the diploma and bachelor's programmes and the factors which contributed to confidence have been presented elsewhere (25).

### Design

A cross-sectional survey design was used for the study. The Government of Gujarat gave permission to perform the study. Appointments for data collection were taken from the principals of the selected institutions through letters. The students were assured of confidentiality, were free to refuse participation or leave midway if they so wished, and were assured that their responses would not influence their final results.

### Setting

This study was carried out in Gujarat situated in the northwest of India. The maternal mortality ratio of Gujarat is lower, 122 per 100,000 live births, compared to the whole of India, with 178 per 100,000 live births (26). The proportion of institutional births in Gujarat is 78%, higher than in India as a whole at 73%. Eighty-five per cent of births in Gujarat are attended to by SBAs compared to 76% in India as a whole (27).

There are 2,670 diploma schools and 1,578 bachelor's colleges of nursing in India out of which more than 90% are private institutions (28). Gujarat had 134 educational institutions for nursing and midwifery at the time of this study. Out of 92 diploma schools, 71 (77%) and out of 42 bachelor's colleges 34 (81%) were privately owned. According to the Indian Nursing Council, it is mandatory for each student to attend atleast 35 births for securing a diploma and 20 for a bachelor's degree, along with other procedures such as episiotomies.

### Sample

From a list of 134 institutions provided by the Gujarat Nursing Council, 79 were excluded as they were newly established and therefore did not have final-year students. The remaining 55 institutions were divided into four strata according to their ownership (government or private) and the type of programme (bachelor's or diploma). We aimed to select 30% of students from each stratum. We picked names of institutions randomly until we could get at least 45% of students from each stratum (considering the possibility of dropouts). Twenty-five institutions were thus selected: 17 schools (out of 38) and 8 colleges (out of 17). All final-year students from these institutions participated in the study.

### Data collection

A questionnaire was designed for the study. The list of skills under four selected ICM competency domains was reviewed by a group of six senior midwifery teachers rating each skill statement on a three-point scale, for relevance to the Indian context and technical terms. The content and language of the ICM was retained as far as possible. Skills that were out of scope of nurse-midwives practice in India were excluded (such as ‘using Doppler to monitor foetal heart rate’). Skill statements, which included more than one skill, were divided into separate statements.

The questionnaire was translated into Gujarati and pilot tested with both the bachelor's (*n=*9) and diploma students (*n=*15), for understanding of statements and the response time. Background questions such as age, sex, religion, and qualification on admission were included as well as total number of births attended. Self-confidence was assessed on a four-point scale – ‘I do not have skill’ (1), ‘I have little skill but need a lot of practice’ (2), ‘I have some skill but need some more practice’ (3), and ‘I am confident’ (4) – and was summed to produce a total score. The question read as ‘If you are posted in a day and night primary health centre where there is no doctor how confident are you to perform this skill’? The data were collected between February and July 2013 to coincide with the term ending. The students answered the questionnaires independently, but the first author was available to clarify any items. However, the presence of the first author could be the reason for few (0.1 to 4%) missing data.

### Statistical methods

The Statistical Package of Social Science (SPSS) Version 20 (SPSS, Inc., Chicago, USA) was used for managing and analysing data. First principal component analysis (PCA) with oblimin rotation (29) was performed separately for the four domains to identify subscales and to group/reduce the number of statements. The factorability of data was assessed through the Kaiser–Meyer–Olkin measure of sampling adequacy (KMO), which should be above 0.6 (30), and Bartlett's test of sphericity (31) to be significant (*p*≤0.05). The KMO values were 0.92–0.95 for the four domains (antepartum, intrapartum, postpartum, and newborn care), all exceeding the recommended value of 0.6 (30), and Bartlett's test of sphericity (31) reached statistical significance in all domains, supporting the sample adequacy and the factorability. The number of retained subscales was guided by Kaiser's criterion (eigenvalues>1), and Cattell's scree test (32) with inspection of the scree plot. All components with an eigenvalue >1 and statements loading above 0.40 were retained. The internal consistency reliability for each of the subscales was measured using a Cronbach's alpha coefficient (33).

### Subscales for antepartum care

Three subscales were identified for the domain of antepartum care. Each subscale explained 38, 8, and 7% of the variance, respectively, and 53% of the total variance, and were labelled as:

1. *Assess maternal and foetal health* contained six skills: a) take antenatal history, b) make a physical examination of the mother, c) Perform complete abdominal assessment; measure fundal height, lie, position, presentation, d) assess foetal growth by manual measurements, e) interprete foetal health and activity, f) explain to the mother the findings of assessment.
2. *Identify antepartum risks* contained four skills: a) identify symptoms of risk, b) take up first-line management of high-risk pregnancies based on national and local guidelines, c) administer life-saving drugs, and d) maintain records.
3. *Provide counselling and health education to women and families* contained nine skills: a) take maternal vital signs such as blood pressure and b) interpret them, c) communicate with the mother about her vital signs, d) advise on expected delivery date, e) assess mothers’ nutritional status and f) educate her on nutrition, g) guide her concerning common discomforts, and h) guide her for birth preparation, i) Provide health education to adolescents, women & families about normal pregnancy progress, danger signs & symptoms.

### Subscales for intrapartum care

The four subscales identified for intrapartum care explained 38, 7, 5, and 4% of the variance, respectively, and 53% of the total variance. They were labelled as:

1. *Manage and monitor the first stage of labour* included eight skills: a) take history and vital signs, b) complete a physical examination, c) complete a vaginal examination, d) monitor uterine contractions, e) assess uterine contractions, f) use the partograph, g) identify abnormal labour patterns for taking action, and h) encourage normal labour.
2. *Manage the second- and third-stages of labour and its complications* included 18 skills: a) augment and b) monitor labour (2 skills), c) perform version, d) administer anaesthesia to the perineum, e) perform episiotomies and f) repair tears (2 skills), g) actively manage third-stage labour including h) use of uterotonic drugs (2 skills), i) manage cord around baby's neck, j) give fundal massage, k) inspect the placenta and membranes, l) assess for perineal and cervical lacerations and tears and m) take action (2 skills), n) manage postpartum bleeding, o) perform aortic compression, p) manually remove the placenta, q) identify and manage shock (2 skills), and r) provide immediate life-saving interventions in emergencies including timely referral.
3. *Perform routine procedures during labour* included five skills: a) administer prescribed drugs as per guidelines, b) insert intravenous line, c) draw blood for laboratory, d) provide environment for mother and child bonding immediately after birth, and e) manage documentation.
4. *Support women during labour* included four skills: a) provide physical and psychological support for the woman and her family, b) promote normal birth, c) facilitate the presence of birth companion, d) ensure hydration and nutrition of mother during labour, and e) provide choices to mother during labour.

### Subscales for postpartum care

Two subscales identified under the domain of postpartum care explained 47.8 and 10.4% of the variance, respectively, and 58% of the total variance and were labelled:

1. *Postpartum physical examination and treatment* included six skills: a) perform physical examination of mother, b) assess uterine involution and healing of lacerations, c) provide contraceptive services, d) detect and manage complications before referral, e) provide emergency treatment for late postpartum haemorrhage, and f) support families in case of bereavement and loss.
2. *Postpartum health education and information to parents* included seven skills: a) take a routine history of the birth; b) support immediate breastfeeding; c) educate mothers for self-care and care of the newborn at home; d) educate the mother about contraception, e) nutrition, f) hygiene, signs of infections, g) rest, and exercises.

### Subscales for newborn care

Three subscales identified for newborn care explained 50, 8.6, and 6.5% of the variance, respectively, and 64.5% of the total variance. They were labelled as:

1. *Initiate essential newborn care* included five skills: a) cutting and clamping cord, b) drying, clearing airways, and establishing respiration, c) assessing the immediate condition of the newborn, d) maintaining body temperature (skin to skin included), e) caring for low birth weight newborn and initiating breastfeeding.
2. *Identify and treat newborn complications* included six skills: a) Begin emergency measures for respiratory distress, b) for hypothermia, c) for hypoglyceamia, d) identify complications for low birth weight infants and refer, e) Screen for congenital defects, f) Transfer at-risk newborns to emergency care facility.
3. *Educate parents about newborn care* included seven skills: a) Educate parents for normal growth and development of infant its day to day needs, b) Assist parents in accessing community resources for care. Support parents in case of c) loss of pregnancy or stillbirth, d) separation from sick newborn, e) multiple babies, f) HIV positive mother, g) document and maintain records.

Medians and percentiles were calculated to assess confidence for the identified subscales for the four domains of the ICM competencies. The results are presented for three groups (0–50 percentile, 50–75 percentile, and >75 percentile).

### Findings

Ninety-eight per cent of the students from the selected institutions participated, as 2% were absent on the day of data collection. From the total 1,525 final-year students available in the state at the time of data collection, we could cover 633 (41%) students (Table 1).

**Table 1:** Sample distribution across categories of institutions

	Diploma		Bachelor		Total	
			
Type of institution	Available	Selected	Available	Selected	Available	Selected
Government	547	223 (40.8%)	185	85 (45.9%)	732	308 (42%)
Private	373	141 (37.8%)	420	184 (43.8%)	793	325 (41%)
Grand total	920	364 (39%)	605	269 (44.5%)	1,525	633 (41.5%)

We could get proportionately 40–45% of students from each stratum (Table 1). The variations in proportions of students selected from each stratum reflect the variations found in class size ranging from 15 to 65.

The median age of the students was 21 years, ranging from 20 to 23 years, 93% were females, 92% were unmarried, and 91% were Hindus. Sixty-four per cent of the students said nursing education was their first choice, whereas 8% preferred to work as midwives after their graduation.

### Antepartum care

**Fig. 1:**
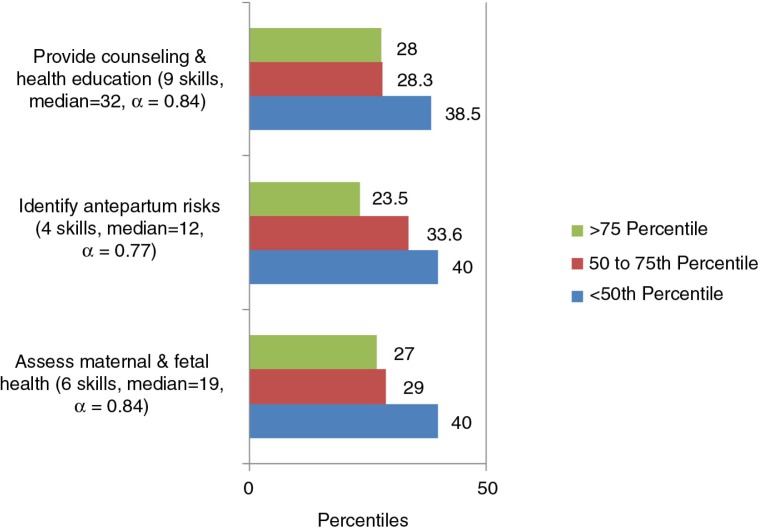
Confidence by percentiles for the subscales identified through PCA for antepartum care (19 skills).

Almost 40% of the students scored below the 50th percentile of confidence, whereas between 23 and 28% were above the 75th percentile for the three antepartum subscales (Fig. 1).

From the 19 skills statements under the antepartum domain, more than 20% of the students expressed low confidence in: assessing foetal growth through abdominal palpation (23%), interpreting foetal heart rate assessment (20%), initiating first-line management of high-risk factors based on national guidelines, and administering prescribed life-saving drugs (38%).

### Intrapartum care

**Fig. 2:**
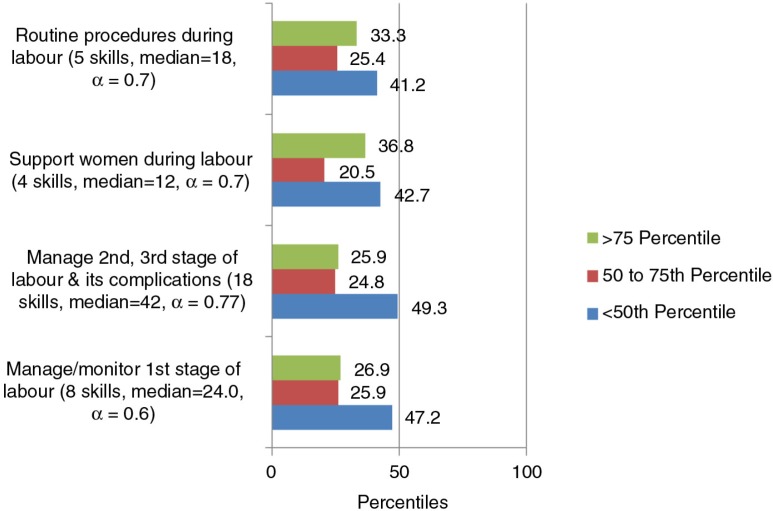
Confidence by percentiles for the subscales identified through PCA for intrapartum care domain (35 skills).

There were 41–49% students below the 50th percentile and 26–33% above the 75th percentile of confidence in the four subscales of intrapartum care (Fig. 2).

Except for three skills statements, more than 20% of the students expressed low confidence in all other skills under intrapartum care. For instance, students expressed low confidence in physical examination during labour (21%), performing per vaginal examination during labour (41%), and monitoring the time of uterine contractions and assessing their effectiveness (33–37%). The students expressed low confidence in active management of third stage of labour (47%), administering local anaesthesia to perineum, and performing episiotomy (56–63%). About 42% had low confidence in identifying abnormal labour patterns, and 53% in providing life-saving measures before referral. About 54% had low confidence in managing cord around baby's neck, 61% in repairing tears and cervical lacerations, 53% in managing postpartum haemorrhage, 57% in manual removal of placenta, and 53% in identifying and managing shock. Eighty-seven per cent had low confidence in aortic compression.

### Postpartum care

**Fig. 3:**
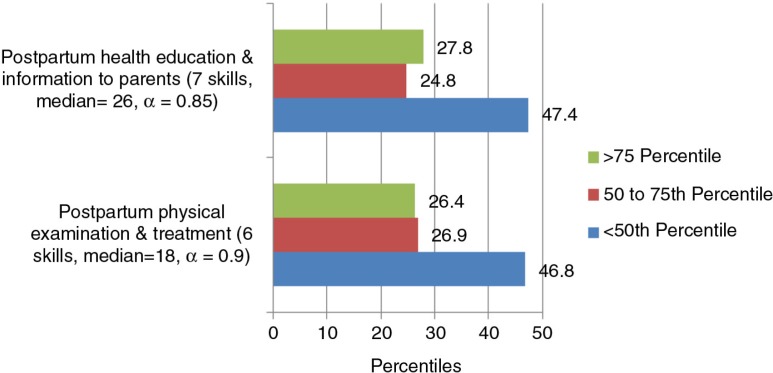
Confidence by percentiles for the subscales identified through PCA for postpartum care (13 skills).

Between 46 and 47% students were below the 50th percentile and 27% were above the 75th percentile of confidence for both the subscales of postpartum care (Fig. 3).

The students expressed low confidence in assessing uterine involution and healing of lacerations and tears (31%), providing first-line treatment for complications and timely referral (32%), and in managing late postpartum haemorrhage (47%). Seventeen per cent of the students expressed low confidence in supporting bereaved families and providing postpartum family planning care.

### Newborn care

**Fig. 4:**
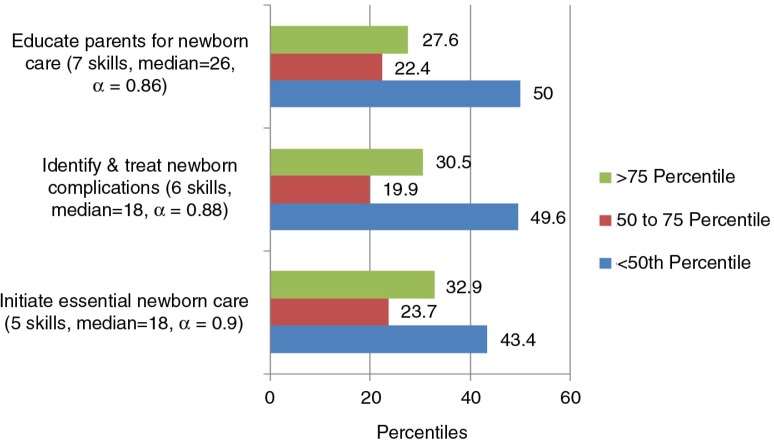
Confidence by percentiles for the subscales identified through PCA for newborn care domain (18 skills).

There were 43–50% students below the 50th percentile for the subscales of newborn care (Fig. 4).

Students expressed low confidence in providing essential newborn care according to national guidelines (16%), in managing newborn complications such as hypothermia and respiratory distress and managing referral for such babies (29–40%), and in screening for congenital anomalies and supporting parents with babies who were HIV positive (15–20%).

### Births attended

**Fig. 5:**
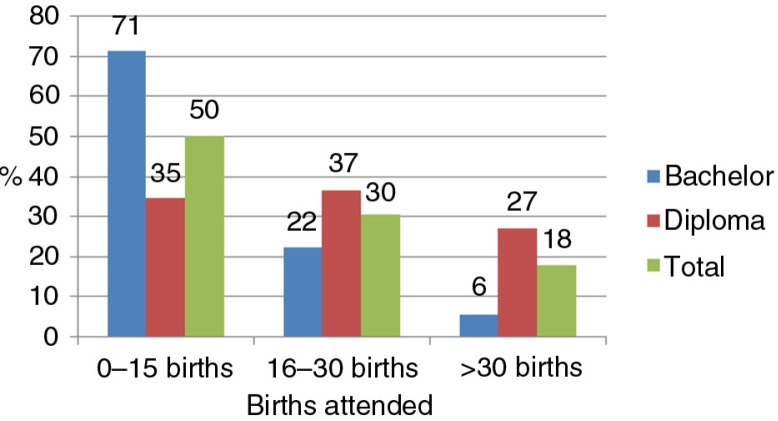
Total number of births attended by type of programme.

The INC requires each student to attend at least 30 births for registration as a midwife. Twenty-six per cent of the diploma students and 15% of the bachelor's students reported attending the required births for midwife registration, whereas 18% of students exceeded the minimum requirements. Fifty per cent students reported attending 0–15 births, and 30% reported attending between 16 and 30 births (Fig. 5).

## Discussion

This study shows that nearly half of the students from both the pre-service education programmes had confidence below the 50th percentile in all the subscales under the four selected domains of midwifery competencies. In the antepartum and postpartum care domains, the students expressed low confidence in recognizing and managing complications before referral. For the newborn care domain, 30–40% students expressed low confidence in handling newborn complications but 16% students also expressed low confidence in essential newborn care which is a basic skill set. It is alarming that in the intrapartum care domain, about 30–40% students expressed low confidence in performing even basic skills such as per vaginal examinations, and counting and assessing uterine contractions. More than half of the students expressed low confidence in recognition and management of intrapartum complications.

There could be several factors which contributed to the overall low confidence levels amongst graduating students. Brown and colleagues (2003) found age as an important variable influencing confidence (34). Analysing for age was not possible for this study because little variation was found in the age of participants. The student participants from this study were young and, therefore, likely to be low in confidence for life in general. Attending childbirth, unlike all other fields of nursing, requires more autonomy and therefore taking responsibility of the well-being of the mother and the baby. The way the question on confidence was framed in this study, ‘performing this skill in a day and night primary health centre where there is no doctor’, could have intimidated them and influenced their responses.

Studies have also found that the lower levels of confidence of graduating midwives and nurses improves after 6 months or a year of work experience (35, 36). It was difficult to include re-assessment of confidence in this study as there is no system to track the place of posting of the graduates. Also, only a few would be posted in the maternity sections of hospitals.

The current study also finds that the students have reported having less clinical hands-on practice, in terms of number of births attended. This possibly is a major contributor to low confidence even in basic skills, such as providing essential newborn care, and many basic intrapartum care skills. Similar findings have been reported by Chaturvedi and colleagues (37), who assessed the skills of practicing staff nurses (SBAs) in emergency obstetric care. They found 14% of respondents were competent at assessment, 58% were competent at making a correct clinical diagnosis, and 20% were competent at providing first-line care. They also found that the SBA training made only a marginal difference to their scores. Therefore, the reservations expressed by professionals about the inadequacy of 2- to 3-week in-service SBA training for staff nurses being implemented by the Government of India are not unfounded. The study by Jishnu Das and colleagues (38), in the district of Madhya Pradesh in India also showed poor skills of health service providers and one factor was the quality of their medical education.

The INC issues a license for midwives. However, midwifery in India is unregulated in terms of a clear definition of scope of midwifery practice (39), which is also reflected in midwifery education. Like many other parts of Southeast Asia (40), India does not have national standards for midwifery education and lacks accreditation systems to monitor the quality of education to ensure that students being awarded the midwifery license are competent and fit to practice.

India is amongst the 43% middle- and low-income countries found by the survey of ICM and the United Nations Population Fund to have an integrated pathway for nursing and midwifery, the other pathways being direct entry midwifery education and midwifery education post-nursing (8). According to the World Health Organization and ICM, integrated programmes are the least effective as they compromise development of competencies for both the nursing and the midwifery professions (41). In the bachelor's programme in India, 11.6% of the total time is allocated to midwifery, and 18.6% of the total time is allocated in the diploma programme. This works out to be about 5–6 months in the 3- to 4-year programmes, falling short of the WHO and ICM recommendations of the duration required to develop competent midwives who could provide comprehensive maternal and newborn services (42). Historically, in pre-independent India and almost a decade after independence, midwives were distinct from nurses. Both the professions got integrated somewhere around 1957 for reasons unclear (43).

Confidence is associated with the learning environment (20). Further research is needed to understand the basic structure, content, and pedagogical approaches used for midwifery education such as structured learning experiences for students, systems for clinical supervision and mentorship, and existing methods of assessment of student's clinical skills for both the programmes. The overall lack of confidence amongst students seen in this study could be seen as an indicator of their future performance as midwives because self-confidence has a mediating effect on performance and could be one of its predictors (16).

### Methodological considerations

The sample size for the current study was large enough to be able to use factor analysis for reduction of data and therefore enable further analysis to get clear and specific findings. The questionnaire prepared for the study included skill statements taken from the list of midwifery competencies of the ICM (7). This is a strength of the study because the list of competencies has been prepared using the Delphi technique, in which experts from 52 countries participated (6). Six midwifery teachers from India reviewed the competencies for their relevance to the Indian context which further validated the questionnaire.

The confidence of students is not measured on the full set of competencies required for a fully qualified midwife as defined by ICM. Only four ICM domains out of seven and skills are included leaving out the ‘knowledge’ and ‘professional behaviour’ (for the practical reasons of reducing the tool length). Because the skills included in the study are ‘basic or core, i.e. those that should be the minimum expected outcome of midwifery pre-service education’ (8), it is reasonable to expect that all graduating students should have confidence in at least these core skills.

Another limitation of the study is using the 75th percentile to categorize high and low confidence, which makes assessment relative to the group participating, rather than a standard. However, we could not find any standard which could be used for assessing levels of confidence against each competency domain.

## Conclusions

The students from the diploma and bachelor's programmes in Gujarat do not feel confident in core midwifery skills listed by the ICM. Though there could be other factors which contribute to low levels of confidence, the study indicates lack of hands-on experience as one of the important factors. The duration, content, and pedagogy of midwifery education within the integrated nursing and midwifery programmes in India need to be reviewed. This study successfully informs the policymakers in charge of maternal and newborn health, and teachers of midwifery, about the specific skills where students need to focus.

The study further indicates that India needs to review the effectiveness of its policy of integrated pre-service education for nurses and midwives. Is it efficient to educate all nurses with some midwifery skills? Or is it better to educate a few with comprehensive skills?

Given the still high maternal and neonatal mortality in India, and India's commitment to ensure skilled care at every birth, the current study is an important contribution towards developing an effective policy to scale up midwifery services in India. It appears to be the first study related to confidence in midwifery skills of graduates to be licensed as midwives.
